# Adaptive Laboratory Evolution Restores Solvent Tolerance in Plasmid-Cured Pseudomonas putida S12: a Molecular Analysis

**DOI:** 10.1128/AEM.00041-21

**Published:** 2021-04-13

**Authors:** Hadiastri Kusumawardhani, Benjamin Furtwängler, Matthijs Blommestijn, Adelė Kaltenytė, Jaap van der Poel, Jan Kolk, Rohola Hosseini, Johannes H. de Winde

**Affiliations:** a Institute of Biology Leiden, Leiden University, Leiden, The Netherlands; University of Helsinki

**Keywords:** adaptive laboratory evolution, solvent tolerance, industrial biotechnology, genome engineering, RND efflux pump

## Abstract

Pseudomonas putida S12 is inherently solvent tolerant and constitutes a promising platform for biobased production of aromatic compounds and biopolymers. The megaplasmid pTTS12 of P. putida S12 carries several gene clusters involved in solvent tolerance, and the removal of this megaplasmid caused a significant reduction in solvent tolerance. In this study, we succeeded in restoring solvent tolerance in plasmid-cured P. putida S12 using adaptive laboratory evolution (ALE), underscoring the innate solvent tolerance of this strain. Whole-genome sequencing identified several single nucleotide polymorphisms (SNPs) and a mobile element insertion enabling ALE-derived strains to survive and sustain growth in the presence of a high toluene concentration (10% [vol/vol]). We identified mutations in an RND efflux pump regulator, *arpR*, that resulted in constitutive upregulation of the multifunctional efflux pump ArpABC. SNPs were also found in the intergenic region and subunits of ATP synthase, RNA polymerase subunit β′, a global two-component regulatory system (GacA/GacS), and a putative AraC family transcriptional regulator, Afr. Transcriptomic analysis further revealed a constitutive downregulation of energy-consuming activities in ALE-derived strains, such as flagellar assembly, F_o_F_1_ ATP synthase, and membrane transport proteins. In summary, constitutive expression of a solvent extrusion pump in combination with high metabolic flexibility enabled the restoration of the solvent tolerance trait in P. putida S12 lacking its megaplasmid.

**IMPORTANCE** Sustainable production of high-value chemicals can be achieved by bacterial biocatalysis. However, bioproduction of biopolymers and aromatic compounds may exert stress on the microbial production host and limit the resulting yield. Having a solvent tolerance trait is highly advantageous for microbial hosts used in the biobased production of aromatics. The presence of a megaplasmid has been linked to the solvent tolerance trait of Pseudomonas putida; however, the extent of innate, intrinsic solvent tolerance in this bacterium remained unclear. Using adaptive laboratory evolution, we successfully adapted the plasmid-cured P. putida S12 strain to regain its solvent tolerance. Through these adapted strains, we began to clarify the causes, origins, limitations, and trade-offs of the intrinsic solvent tolerance in P. putida. This work sheds light on the possible genetic engineering targets to enhance solvent tolerance in Pseudomonas putida as well as other bacteria.

## Abstract (precis)

Sustainable production of high-value chemicals can be achieved by bacterial biocatalysis. However, bioproduction of biopolymers and aromatic compounds may exert stress on the microbial production host and limit the resulting yield.

## INTRODUCTION

Pseudomonas putida is a promising microbial host for biobased production of valuable chemicals and biopolymer compounds (1). Endowed with a natural versatility, P. putida is robust toward toxic compounds which may arise in whole-cell biocatalysis processes as substrates, intermediates, or products. P. putida displays a remarkable intrinsic oxidative stress and solvent tolerance. This may be further optimized for utilization of secondary feedstock as a carbon source and production of various aromatic compounds and bioplastic monomers (2–9). Moreover, several metabolic models and genetic tools are currently available for the design and implementation of novel biosynthetic pathways in P. putida (10–12).

P. putida S12 was isolated from soil on minimal medium with styrene as its sole carbon source (13). This strain has been used to produce a variety of high-value aromatic compounds (6, 8, 9, 14). Organic solvents and aromatic compounds are toxic to most bacteria, as these compounds are able to accumulate in the bacterial membrane and thus alter membrane integrity (15), resulting in damage and loss of various membrane functions, such as permeability barrier, scaffold for membrane-bound protein and metabolic reaction, energy transduction, and denaturation of essential enzymes. Solvent-tolerant bacteria, like P. putida S12, are able to mitigate such damage by extruding organic solvent molecules and changing their membrane composition to prevent solvent accumulation in the membrane (15–17).

The P. putida S12 genome comprises a 5.8-Mbp chromosome and a single-copy 583-kbp megaplasmid, pTTS12 (18). Plasmid pTTS12 encodes, among others, an RND efflux pump (SrpABC), a styrene-phenylacetate degradation pathway, and a toxin-antitoxin module, SlvTA, which are responsible for the high solvent tolerance of P. putida S12 (18, 19). A significant reduction of solvent tolerance was previously demonstrated when P. putida strains were cured from its megaplasmid (19, 20). As a result of plasmid curing, P. putida S12 ΔpTTS12 could survive and sustain growth only in a maximum of 0.15% (vol/vol) toluene (19). As a comparison, wild-type S12 can sustain growth in 0.30% (vol/vol) toluene and survive in up to 10% (vol/vol) toluene. However, as was previously demonstrated, the expression of the SrpABC efflux pump in the non-solvent-tolerant Escherichia coli strains instigated a lower solvent tolerance than in P. putida S12, demonstrating that other genes contribute to the solvent tolerance phenotype of P. putida S12 (19).

In this paper, we further addressed the innate, intrinsic solvent tolerance of P. putida S12. Megaplasmid pTTS12 may confer genetic adaptation toward environmental chemical stressors like organic solvents and aromatic compounds through horizontal gene transfer. Here, we examined the ability of plasmid-cured P. putida S12 to survive and sustain growth in the presence of toluene. Using adaptive laboratory evolution (ALE), we were able to restore the solvent tolerance in P. putida S12 lacking the megaplasmid. Specific mutations putatively responsible for the restored solvent tolerance trait were characterized. Moreover, transcriptome analysis (RNA-seq) revealed the constitutive responses of plasmid-cured P. putida S12 after adaptation to the elevated toluene concentration.

## RESULTS

### Plasmid-cured Pseudomonas putida S12 can regain the ability to tolerate high-concentration toluene.

**FIG 1:**
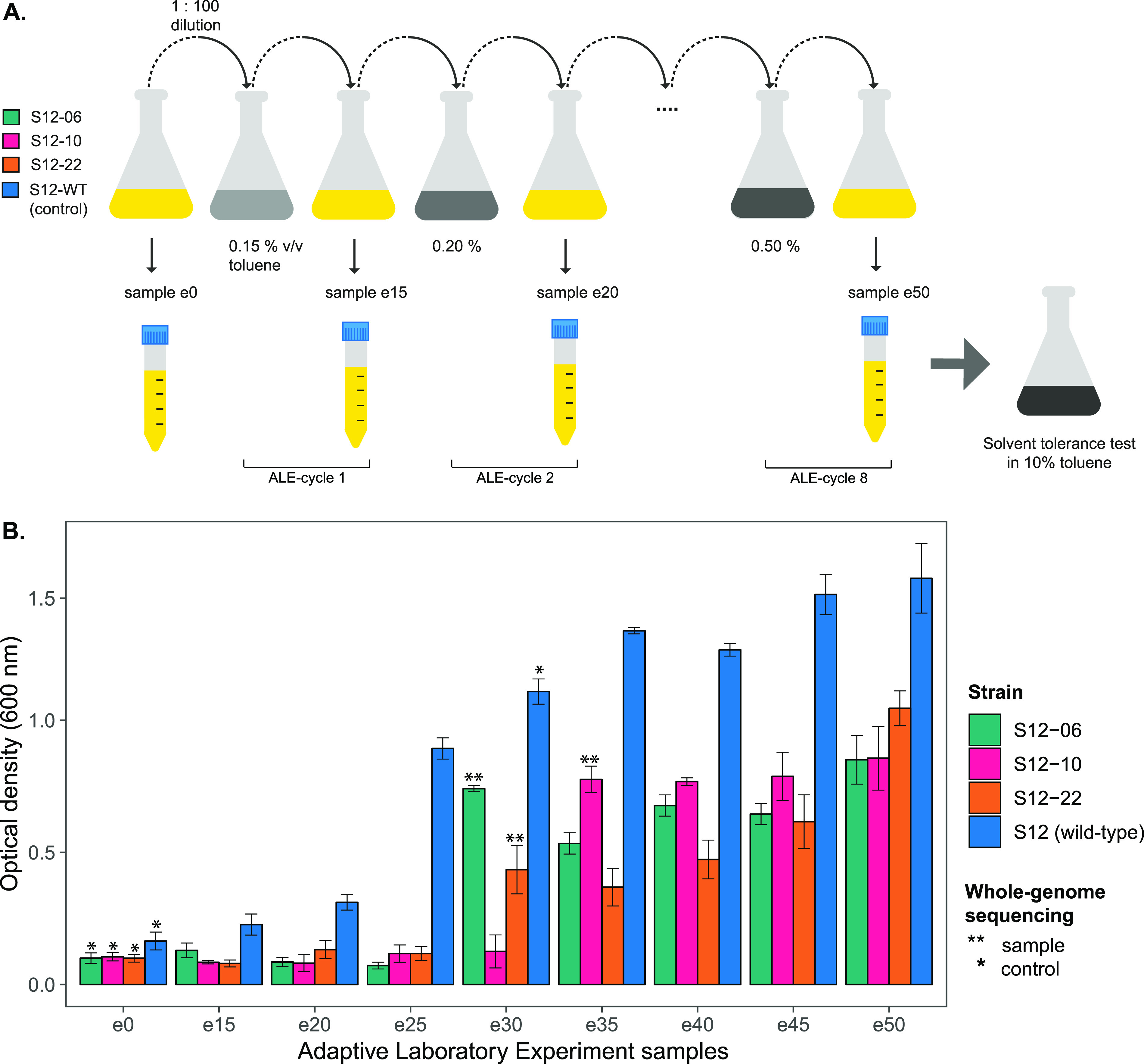
Adaptive laboratory evolution (ALE) experiment of plasmid-cured P. putida S12 to increasing concentrations of toluene. (A) Experimental design of ALE. ALE was performed on three plasmid-cured P. putida S12 strains (S12-06, S12-10, and S12-22). In the ALE experiment, LB medium (yellow) was used as the growth medium with the addition of an increasing toluene concentration of 0.05% (vol/vol) every cycle (gray). (B) Plasmid-cured P. putida S12 regained the ability to grow in high toluene concentrations. The solvent tolerance phenotype of ALE-derived strains was tested by observing strain growth on LB medium with 10% (vol/vol) toluene within 48 h. Single and double asterisks indicate the control and sample strains, respectively, that were taken for whole-genome sequencing. This experiment was performed with three biological replicates, and error bars indicate standard deviations.

To investigate the intrinsic solvent tolerance of P. putida S12, we performed an adaptive laboratory evolution (ALE) experiment on plasmid-cured P. putida S12. Three biological replicates of plasmid-cured P. putida S12 (strains S12-06, S12-10, and S12-22) and a wild-type P. putida S12 as the control were set up to grow on lysogeny broth (LB) medium with the addition of 0.15% (vol/vol) toluene, the initial maximum concentration that can be tolerated by plasmid-cured P. putida S12 (Fig. 1A). At stationary phase (typically after 24 to 48 h), these cultures were transferred (1:100 dilution) to grow overnight on fresh LB medium. Overnight LB medium cultures were transferred into LB medium containing 0.20% (vol/vol) toluene (increase of 0.05% [vol/vol] toluene) to continue with the next ALE cycle. While plasmid-cured P. putida S12 is unable to grow on LB medium with 0.20% (vol/vol) toluene directly, these cultures are able to grow on LB medium with 0.20% (vol/vol) toluene after adapting to LB medium with 0.15% (vol/vol) toluene. We repeated this growth cycle with an increasing concentration every cycle until plasmid-cured P. putida S12 strains were able to grow on LB medium with 0.50% (vol/vol) toluene (Fig. 1A). All samples from every ALE cycle were collected and tested for their ability to survive 10% (vol/vol) toluene on LB medium for 48 h. This concentration was chosen to represent a high toluene concentration that creates a distinct second-phase layer in the culture medium.

Initially, plasmid-cured P. putida S12 strains did not show growth or survival in the presence of 10% (vol/vol) toluene, while the wild-type P. putida S12 could survive [(2.52 ± 0.31) × 10^−2^ survival frequency], although it did not show any growth in 10% (vol/vol) toluene (Fig. 1B). After adaptation to a moderate toluene concentration (0.30 to 0.35% [vol/vol]), plasmid-cured P. putida S12 strains showed a significant increase in their ability to withstand and sustain growth in 10% (vol/vol) toluene (Fig. 1B). ALE-derived strains S12-06e30, S12-10e35, and S12-22e30 were able to grow on LB medium with 10% (vol/vol) toluene, reaching final optical densities at 600 nm (OD_600_) of 0.741 ± 0.02, 0.776 ± 0.08, and 0.434 ± 0.158, respectively, after 48 h. These three samples were taken for whole-genome sequencing to map the occurring mutations important for the solvent tolerance phenotype. Wild-type P. putida S12, S12e30, and the initial plasmid-cured P. putida S12 strains were sequenced as controls.

### Common mutations were identified in solvent-tolerant strains obtained from ALE.

**FIG 2:**
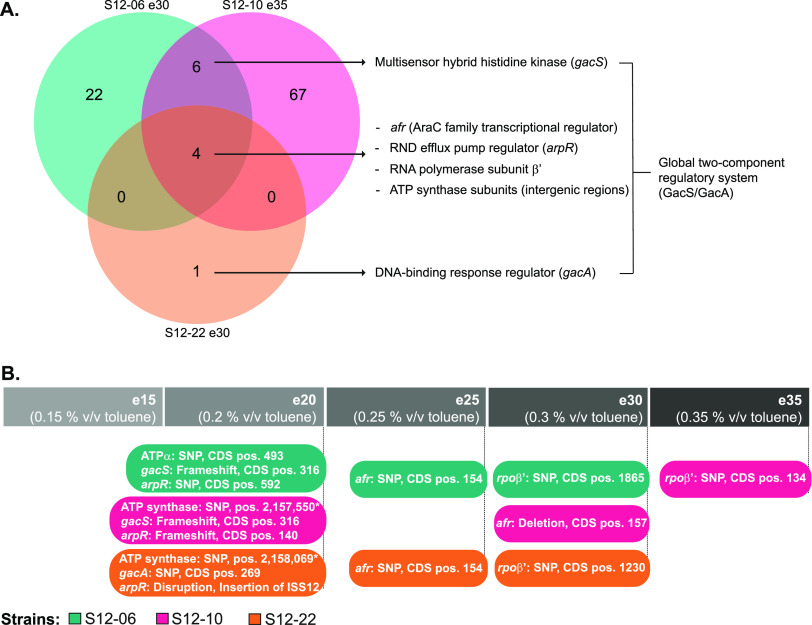
Common mutated loci were identified in the ALE-derived P. putida S12 strains. (A) Venn diagram of mutated loci in the ALE-derived P. putida S12 strains. The colors indicate the three ALE-derived strains. Common mutated loci were identified among ALE-derived strains for an AraC family transcriptional regulator (Afr), RND efflux pump regulator (ArpR), RNA polymerase subunit β’, intergenic region of F0F1 ATP synthase subunits, and global two-component regulatory system GacS/GacA. (B) ALE-derived P. putida S12 strains accumulated key mutations in a stepwise manner. ALE-derived strains were probed for key mutation accumulation by PCR and Sanger sequencing. The colors indicate the three ALE-derived strains, and the gray bar indicates the ALE cycles from which the strains were originating. The positions of the occurring SNPs or indels are indicated as coding DNA sequence (CDS) positions of each mutated locus, except for the SNPs within the ATP synthase intergenic regions, whose positions are indicated relative to the chromosome sequence.

We performed whole-genome sequencing of ALE-derived strains S12-06e30, S12-10e35, and S12-22e30 to map the occurring mutations that may lead to increased solvent tolerance in the evolved strains. We identified 32, 77, and 5 mutations (single nucleotide polymorphisms [SNPs], insertions/deletions [indels], and mobile element IS*S12* insertion), respectively, in S12-06e30, S12-10e35, and S12-22e30 (Fig. 2A and see Table S2 in the supplemental material). Among these mutations, four common mutated loci were identified in all strains. These mutations occurred in the AraC family transcriptional regulator Afr (RPPX_14685), in the RND efflux pump regulator ArpR (RPPX_14650), in RNA polymerase subunit β′ *rpoB*′ (RPPX_06985), and in the intergenic regions and subunits of ATP synthase (RPPX_09480–RPPX_09510) (Fig. 2A and Table S2). Six mutated loci were shared only between S12-06e30 and S12-10e35. Among these six loci, indels occurred within the *gacS* locus (RPPX_15700) in S12-06e30 and S12-10e35, while S12-22e30 had a unique SNP within the *gacA* locus (RPPX_00635). In *Pseudomonas*, GacS and GacA proteins are known to constitute a two-component regulatory system which regulates biofilm formation, cell motility, and secondary metabolism (21).

Since the ALE-derived strains showed a sudden increase in their ability to tolerate high toluene concentrations, we investigated the order of accumulation of key mutations in ALE-derived strains. Key mutations accumulated in a stepwise manner rather than emerging simultaneously in one cycle (Fig. 2B). In the second ALE cycle, three key mutations occurred under exposure to 0.20% (vol/vol) toluene in all strains (S12-06e20, S12-10e20, and S12-22e20). The first accumulated mutations occurred on the intergenic regions between ATP synthase subunits, the *gacS* and *gacA* loci, and the *arpR* locus. In the subsequent cycle, S12-06e25, S12-10e30, and S12-22e25 accumulated additional key mutations in the *afr* locus. The final key mutations on the *rpoB′* locus were accumulated by strains S12-06e30, S12-10e35, and S12-22e30, in which the sudden increase of solvent tolerance was observed.

### Contribution of key mutations to increased solvent tolerance of ALE-derived strains.

**FIG 3:**
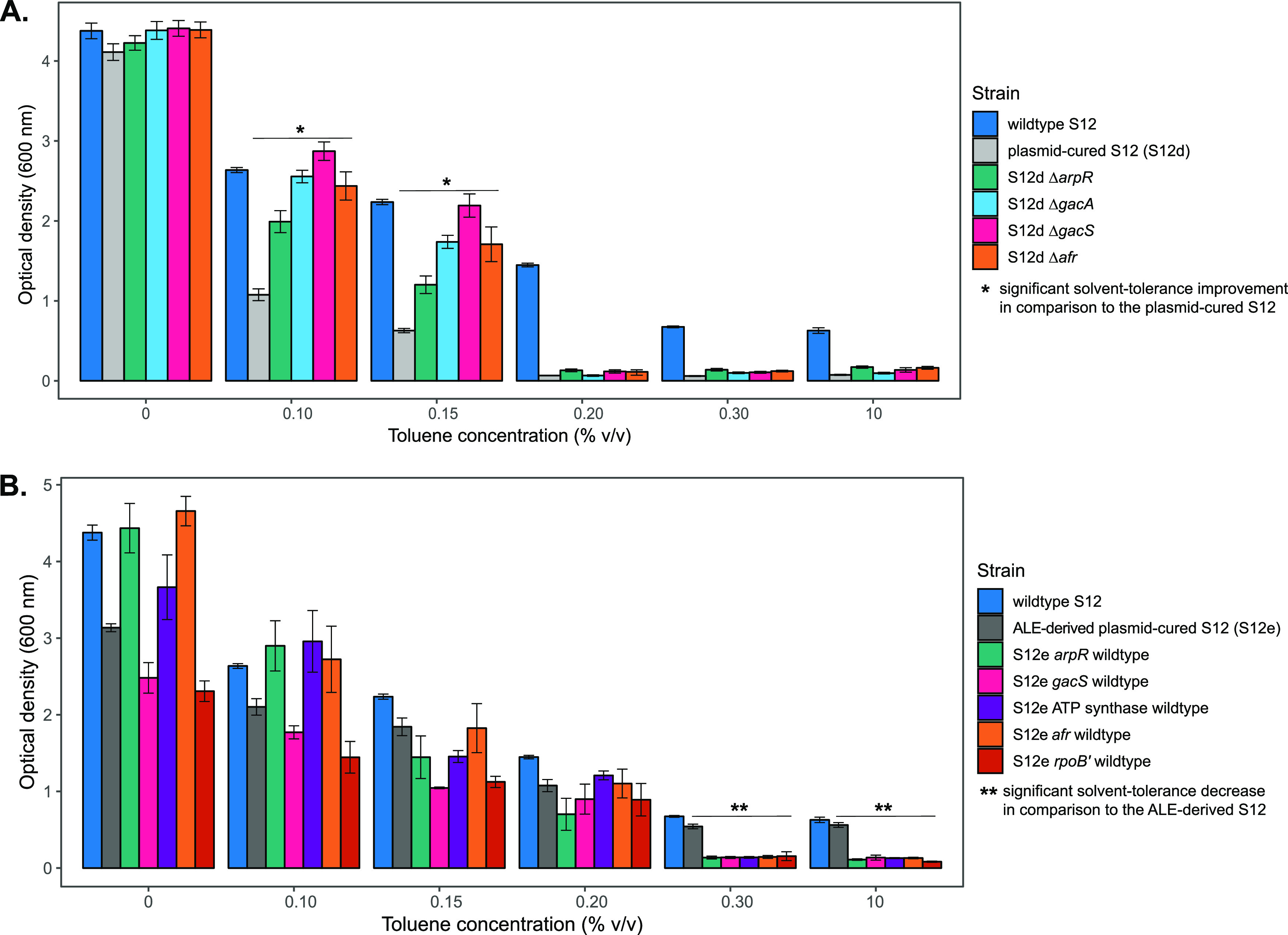
Accumulated key mutations contributed to the solvent tolerance phenotype of ALE-derived P. putida S12 strains. (A) Single knockout of common mutated loci in the plasmid-cured P. putida S12 strain improved growth on LB medium with a low toluene concentration (0.1 to 0.15% [vol/vol]). Different colors indicate the control strains and the plasmid-cured S12 strains with deleted loci. This experiment was performed with three biological replicates, and error bars indicate standard deviations. (B) Single restoration of common mutated loci in the ALE-derived P. putida S12 reduced solvent tolerance phenotype. Different colors indicate the control strains and the ALE-derived S12 strains with restored loci. The restored strains can grow on LB medium with a maximum of 0.20% (vol/vol) toluene. This experiment was performed with three biological replicates, and error bars indicate standard deviations.

To study the contribution and impact of each mutated locus, single-knockout strains of *arpR* (RPPX_14650), *afr* (RPPX_14685), *gacA* (RPPX_00635), and *gacS* (RPPX_15700) were created in plasmid-cured P. putida S12. In the ALE-derived strains, the acquired mutations (indels and mobile element insertion) in *arpR* (RPPX_14650), *afr* (RPPX_14685), and *gacS* (RPPX_15700) caused truncation of the encoded protein, while the SNP in *gacA* (RPPX_00635) caused an amino acid residue change (P90L) (Table S2). The SNPs acquired in ATP synthase and in RNA polymerase subunit β′ loci were not addressed with this single-knockout approach, since knocking out these genes would have deleterious effects. Solvent tolerance analysis of the single-knockout strains indicated that deletion of each of those genes improved the growth of plasmid-cured P. putida S12 strains on LB medium with 0.15% (vol/vol) toluene (Fig. 3A). However, single knockout of these genes did not enable plasmid-cured P. putida S12 strains to grow on a toluene concentration higher than 0.15% (vol/vol).

Individual restoration of common mutated loci in the ALE-derived strains to their wild-type sequence caused these strains to lose the ability to withstand the presence of moderate and high toluene concentrations (0.30% and 10% [vol/vol] toluene) (Fig. 3B). These strains can sustain growth on LB medium with a maximum of 0.20% (vol/vol) toluene. Therefore, we concluded that each of the common mutated loci is important for the solvent tolerance phenotype in ALE-derived strains.

### Reverse engineering of key mutations on plasmid-cured S12 successfully restores solvent tolerance.

To confirm the important contribution of key mutations, we introduced these mutations into a plasmid-cured S12 strain (Table 1) and analyzed the growth parameters of the resulting strains in the presence and absence of toluene (Table 2). Strain S12-10 was chosen to represent plasmid-cured S12 in this experiment due to the lowest amount of background mutation (Table S1). It is interesting to note that strain RE2 exhibited significantly better growth parameters in both LB and minimal media than its parent strains RE1 and S12-10 (Table 2). In contrast, the introduction of a third mutation at the ATP synthase subunit alpha (RPPX_09510) in strain RE3 caused a severe reduction in growth parameters in LB and minimal media (Table 2).

**TABLE 1:**
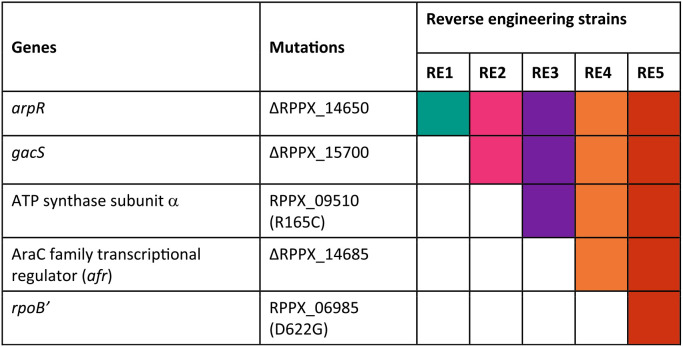
Reverse engineering of the key mutations in plasmid-cured P. putida S12 (strain S12-10)

**TABLE 2:** Growth parameters of ALE-derived and reverse engineering strains^*a*^

Medium and strain	Lag time (min)	μ_max_ (h^−1^)		maxOD	
		Mean	SD	Mean	SD
ALE
LB medium
S12	180	1.18	0.090	0.90	0.038
S12e30	180	1.02*	0.021	0.87	0.027
S12-06	180	1.14	0.026	0.88	0.048
S12-06e30	180	0.80*	0.029	0.82*	0.036
S12-10	180	1.15	0.038	0.89	0.039
S12-10e35	180	0.78*	0.030	0.83*	0.032
S12-22	195	1.07	0.037	0.88	0.045
S12-22e30	180	0.79*	0.018	0.82*	0.034
MM + citrate
S12	180	1.02	0.105	0.88	0.064
S12e30	180	1.05	0.044	0.86	0.027
S12-06	165	1.01	0.095	0.83	0.027
S12-06e30	225	0.48*	0.058	0.54*	0.130
S12-10	165	1.02	0.141	0.83	0.032
S12-10e35	255	0.47*	0.044	0.37*	0.145
S12-22	165	0.99	0.078	0.84	0.019
S12-22e30	240	0.41*	0.049	0.30*	0.115
MM + glucose
S12	150	0.89	0.072	1.00	0.024
S12e30	150	1.06*	0.021	0.99	0.009
S12-06	150	0.95	0.027	1.02	0.033
S12-06e30	180	0.71*	0.028	0.66*	0.074
S12-10	150	0.97	0.058	1.03	0.033
S12-10e35	195	0.70*	0.032	0.70*	0.199
S12-22	150	0.94	0.033	1.02	0.022
S12-22e30	210	0.73*	0.019	0.62*	0.161
MM + glycerol
S12	195	1.16	0.119	1.04	0.006
S12e30	195	1.04	0.010	0.89*	0.046
S12-06	195	1.11	0.138	1.09	0.051
S12-06e30	255	0.54*	0.044	0.82*	0.010
S12-10	180	1.11	0.159	1.08	0.046
S12-10e35	315	0.50*	0.011	0.79*	0.031
S12-22	195	1.17	0.119	1.10	0.044
S12-22e30	300	0.52*	0.029	0.80*	0.011
Reverse engineering
LB medium
RE1	195	0.88	0.013	0.93	0.0498
RE2	105**	0.96**	0.017	0.96	0.0468
RE3	135*	0.88*	0.035	1.07	0.1016
RE4	135	0.89	0.035	1.07	0.0985
RE5	135	0.94	0.029	1.04	0.0937
MM + citrate
RE1	195	1.02	0.048	0.85	0.0605
RE2	150**	0.97	0.025	0.94	0.0460
RE3	240*	0.54*	0.106	0.51*	0.1681
RE4	240	0.57	0.096	0.40	0.0698
RE5	240	0.49	0.067	0.44	0.1328
MM + glucose
RE1	120	0.98	0.031	1.02	0.0326
RE2	120	1.04	0.018	1.00	0.0399
RE3	165*	0.84*	0.077	0.64*	0.0917
RE4	165	0.78	0.083	0.63	0.1286
RE5	165	0.93	0.140	0.59	0.1101
MM + glycerol
RE1	150	1.10	0.044	1.00	0.0958
RE2	120**	1.14	0.072	0.99	0.1264
RE3	180*	0.78*	0.101	1.06	0.2613
RE4	180	0.79	0.098	1.02	0.2867
RE5	180	0.87	0.098	1.10	0.2124

*, impaired growth parameters compared to its parental strain; **, improved growth parameters compared to its parental strain.

**FIG 4:**
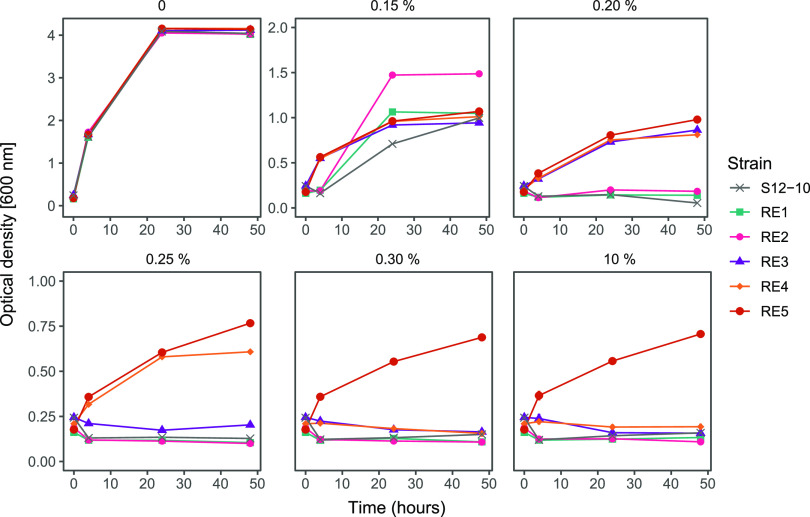
Reverse engineering of the key mutations found in ALE-derived strains. Reverse engineering of the key mutations found in the ALE-derived P. putida S12 strains successfully restored the solvent tolerance phenotype in the plasmid-cured strain S12-10. Different colors indicate the control strain S12-10 and the reverse engineering (RE) strains. This experiment was performed with three biological replicates, and error bars indicate standard deviations. The *y* axis may be different for the panels shown.

Reverse engineering strains and the parent, strain S12-10, were tested for their ability to survive and sustain growth in the presence of toluene (Fig. 4). Strains S12-10, RE1, and RE2 were able to withstand and sustain growth only in the presence of 0.15% (vol/vol) toluene. Nevertheless, strains RE1 and RE2 showed limited growth improvement in comparison to strain S12-10 (Fig. 4, upper middle panel). Strains RE3 and RE4 were able to withstand and sustain growth in slightly higher toluene concentrations, 0.20% and 0.25% (vol/vol), respectively. Finally, strain RE5 was able to sustain growth in the presence of a high toluene concentration (10% [vol/vol]). Taken together with the individual knockouts and restoration of the key mutations (Fig. 3), we can conclude that the actual combination of the key mutations is important for the restoration of solvent tolerance in plasmid-cured S12.

### Restoration of solvent tolerance involved a constitutive downregulation of energy-consuming activities in ALE-derived strains.

Global transcriptional analysis (RNA sequencing) was performed to probe the response of the ALE-derived, wild-type, and plasmid-cured P. putida S12 strains in the presence or absence of toluene (LB medium with 0.1% [vol/vol] toluene). As a response to toluene addition, ALE-derived strains showed differential expression of only 14 loci. This response was in stark contrast to the wild-type S12 and plasmid-cured S12, which differentially expressed more than 500 loci as a response to toluene addition (Fig. S1). Comparison of gene expression between ALE-derived strains with plasmid-cured and wild-type P. putida S12 growing on LB medium in the absence of toluene indicated that the mutations which occurred in the ALE-derived strains caused constitutive differential expression of ±900 genes that play a role in restoring solvent tolerance.

**FIG 5:**
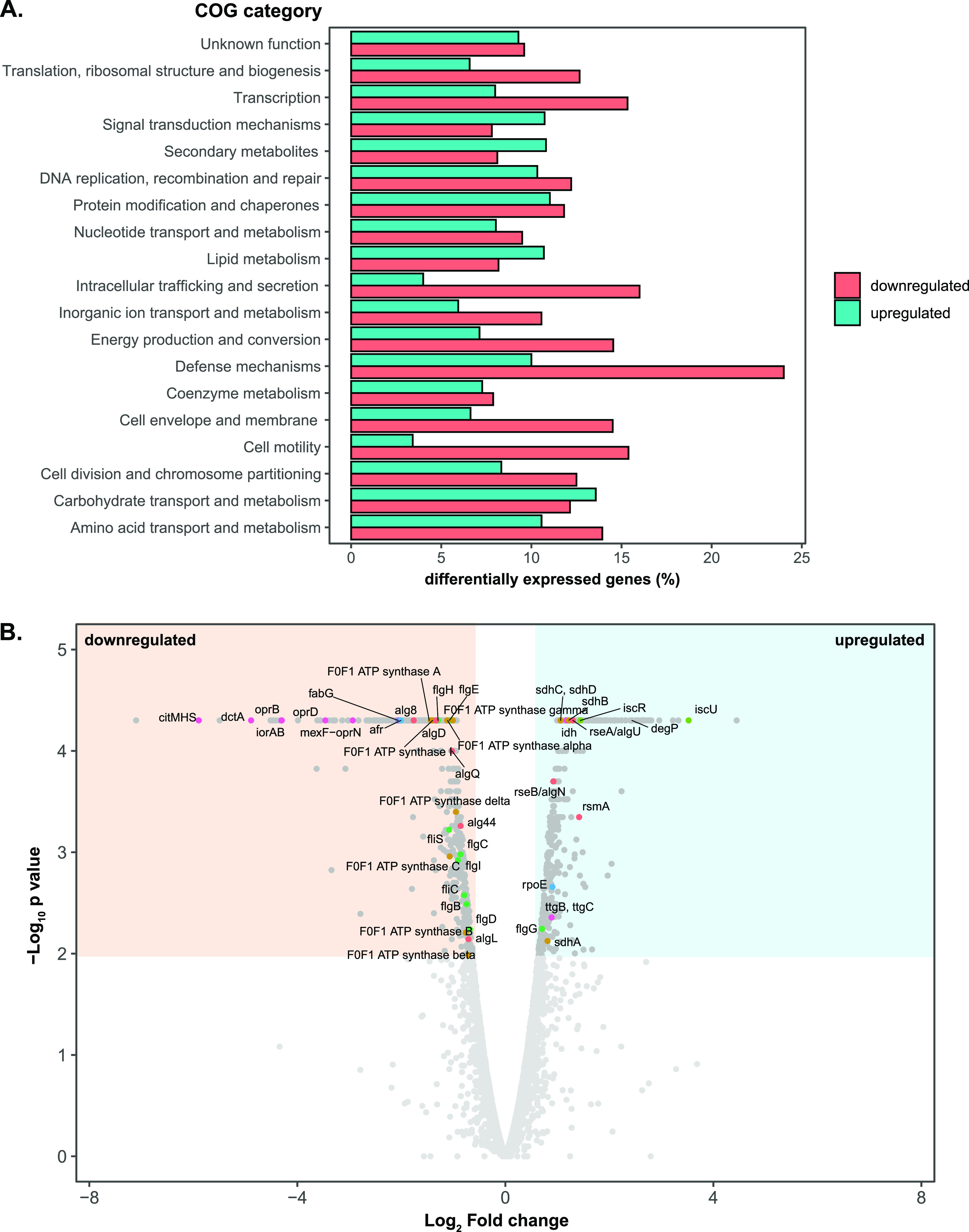
Visualization of differentially expressed genes on ALE-derived P. putida S12 strains in comparison to the parental strain growing on LB medium. (A) COG classification of differential gene expression in ALE-derived strains in comparison to plasmid-cured P. putida S12. COG classification was performed using eggNOG 5.0 mapper (http://eggnogdb.embl.de/#/app/emapper). The percentages of upregulated genes in each class are represented by blue bars, and the percentages of downregulated genes are represented by red bars. (B) Volcano plot of differential gene expression in ALE-derived strains in comparison to the plasmid-cured P. putida S12 growing on LB medium. The blue area indicates the significantly upregulated genes, and the beige area indicates the significantly downregulated genes (cutoff, log_2_ fold change of ≥1 for upregulated genes or ≤1 for downregulated genes and a *P* value of ≤0.01). The colored dots represent the significantly up- or downregulated genes discussed in this paper. Colors correspond to the function of each gene, as follows: red represents biofilm and alginate production genes, orange represents the genes involved in the oxidative phosphorylation process, light green represents the genes involved in the energy production process, green represents flagellar assembly gene clusters, blue represents sigma factor and transcriptional regulator genes, and magenta represents the genes which constitute membrane transporters.

Constitutive differentially expressed genes in ALE-derived strains in comparison to parental plasmid-cured P. putida S12 were classified based on COG categorization. Several classes of genes were downregulated in ALE-derived strains compared to plasmid-cured S12, including, for example, genes constituting cell motility, intracellular trafficking and secretion, and defense mechanism functions (Fig. 5A). In general, ALE-derived strains appeared to constitutively shut down energy-consuming activities, such as flagellar biosynthesis, F_o_F_1_ ATP synthase, and membrane transport proteins which are energized through proton (H^+^) influx. Additionally, genes related to biofilm formation were constitutively downregulated. Here, we focused on several classes of genes that were differentially expressed in ALE-derived strains compared to its parental strain.

### (i) Membrane proteins and efflux pumps.

The ArpABC efflux pump (RPPX_14635–RPPX_14640) is a multifunctional RND efflux pump homologous to TtgABC from P. putida DOT-T1E. This locus was moderately upregulated in ALE-derived strains (Fig. 5B, *ttgB* and *ttgC*). While the upregulation of this pump is a common response to toluene in wild-type P. putida S12, ALE-derived strains constitutively upregulate ArpABC by SNPs and mobile element insertion in its negative regulator gene, *arpR* (RPPX_14650). Interestingly, almost all of the other RND efflux pumps encoded in the chromosome of P. putida S12 were downregulated in ALE-derived strains. In the ALE-derived strains lacking the pTTS12-encoded SrpABC solvent pump, ArpABC is the only remaining efflux pump that may extrude toluene, albeit with a much lower affinity (22). Downregulation of other efflux pumps is likely to be important to preserve the required proton motive force.

Several genes associated with porin function were downregulated in ALE-derived strains, as exemplified by RPPX_10240, RPPX_14820, and RPPX_17640, which encode OprD porin family proteins. This response was similarly observed in a previous proteomics study (23) which noted the downregulation of porins to avoid toluene leakage into the cell through these porins. In addition to porin downregulation, several membrane transport proteins, such as those encoded by *dctA* (H^+^/C_4_-dicarboxylate symporters, RPPX_17630) and *citMHS* (citrate-divalent cation/H^+^ symporter, RPPX_17635), were constitutively downregulated.

### (ii) Energy production and conversion.

In ALE-derived strains, F_o_F_1_ ATP synthase subunits were constitutively downregulated (Fig. 5B). This was in line with our finding of SNPs which occurred on the intergenic regions between F_o_F_1_ ATP synthase subunits (RPPX_09480–RPPX_09510). F_o_F_1_ ATP synthase generates 1 ATP from ADP in bacteria by pumping out 3 H^+^ molecules, and thus downregulation of these loci may also contribute to the preservation of proton motive force.

The succinate dehydrogenase (SdhABCD) gene cluster (RPPX_01070–RPPX_01085) was constitutively upregulated in ALE-derived strains. Succinate dehydrogenase acts as complex II in the oxidative phosphorylation process. Genes coding for cytochrome *c* oxidase subunit II (RPPX_08860) and its assembly protein (RPPX_08850), composing complex IV, were also constitutively upregulated in ALE-derived strains. Taken together, these findings are in line with the importance of the electron transport chain in maintaining proton motive force during solvent stress.

### (iii) Biofilm formation.

In ALE-derived strains, we observed a constitutive upregulation of the *rsmA* locus (RPPX_02245). Upregulation of the *rsmA* locus may be caused by the mutations found in the *gacS* or *gacA* locus and is known to promote a motile lifestyle in *Pseudomonas* (21). Downregulation of the alginate biosynthesis pathway for the main polysaccharide matrix in *Pseudomonas* biofilm was also observed. Alg44 (RPPX_14155), which upon its interaction with c-di-GMP is known to positively regulate alginate production (24), was constitutively downregulated in ALE-derived strains. Other loci which are involved in alginate biosynthesis and export were also downregulated, e.g., *algL* (RPPX_14130), *alg8* (RPPX_14160), *algD* (RPPX_14165), and *algE* (RPPX_21545). Taken together, these findings are indicative of a reduction in biofilm formation capacity in the ALE-derived strains.

**FIG 6:**
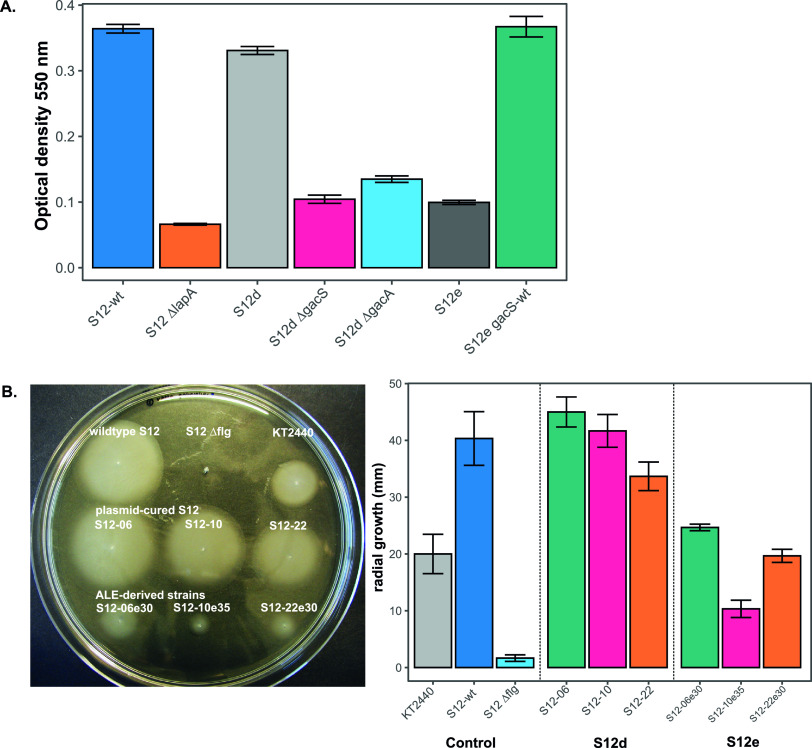
Biofilm formation and cell motility were reduced in ALE-derived P. putida S12 strains. (A) Microtiter biofilm formation assay of P. putida S12. Plasmid-cured P. putida S12 (S12d) Δ*gacS* and Δ*gacA* showed reductions similar to those of ALE-derived P. putida S12 (S12e) strains. Restoration of the *gacS* locus to wild-type sequence (S12e *gacS*-wt) also restored biofilm formation in ALE-derived P. putida S12 (S12e). The measurement of biofilm formation was performed by measuring the optical density at 550 nm, as previously described (25), with the Δ*lapA* (adhesin) mutant taken as a negative control. This experiment was performed with three biological replicates, and error bars indicate standard deviations. (B) Swimming motility assay of P. putida S12 in low-viscosity agar (LB medium plus 0.3% [wt/vol] agar). ALE-derived P. putida S12 strains (S12e) showed a reduced radial growth in low-viscosity agar, indicating lower swimming motility. The Δ*flg* mutant (flagellar gene cluster) was taken as a negative control. In the right panel, the bars represent an average of radial growth of at least three biological replicates of each strain, and error bars indicate standard deviations.

To confirm this result, we applied a microtiter dish biofilm formation assay (25) to assess the biofilm formation in ALE-derived strains (Fig. 6A). Biofilm formation was indeed clearly lower in ALE-derived strains than in wild-type and plasmid-cured P. putida S12 strains. This tendency was reversed when the indel mutation in the *gacS* locus was restored to the wild-type sequence. While biofilm may protect bacteria from external stressors, nutrient and oxygen depletion during a sessile lifestyle may be disadvantageous in solvent stress, and therefore constitutive downregulation of biofilm-related genes was beneficial in ALE-derived strains.

### (iv) Cell motility.

Flagellar biosynthesis loci (RPPX_02045–RPPX_02125) were constitutively downregulated in ALE-derived strains (Fig. 5B). Consequently, this may lead to reduced swimming motility in ALE-derived strains. We confirmed this finding by measuring the radial growth of ALE-derived strains in comparison to that of the wild-type and plasmid-cured P. putida S12 strains on low-viscosity agarose (Fig. 6B). Indeed, ALE-derived strains showed a significant reduction in radial growth. Downregulation of flagella may be a strategy of ALE-derived strains to maintain proton motive force and reroute energy toward extrusion of toluene, since both the RND efflux pump ArpABC and flagella utilize H^+^ influx as an energy source.

### (v) Chaperones.

We also observed the constitutive upregulation of loci RPPX_14680–RPPX_14875, which encode homologs of sigma factor E (RpoE), anti-sigma factor RseAB, and DegP protein, respectively. This cluster is known to orchestrate the expression of chaperone proteins as a stress response to the elevated amount of misfolded proteins in E. coli (26). Additionally, these sigma factors are known to negatively regulate alginate biosynthesis in P. aeruginosa (27). Other chaperone proteins, like Hsp20 protein (RPPX_17155), was also constitutively upregulated in ALE-derived strains. Constitutive upregulation of these genes suggests an important role of chaperones in the adaptive response to high toluene concentrations.

## DISCUSSION

### Solvent tolerance can be restored in a relatively small number of generations.

Damaged macromolecules and cellular components upon exposure to organic solvent may elicit a variety of cellular responses (28, 29). First-line defenses to solvent stress are induced very rapidly following the addition of organic solvent. Membrane compaction and reduction of organic solvent internalization into the cells are examples of such responses (30, 31). However, while such responses ensure the survival of solvent shock, they may not be sufficient to support long-term growth in the presence of solvent. Consequently, in this paper, we investigated other aspects that are required for long-term growth in the presence of high solvent concentrations.

The single-copy megaplasmid pTTS12 plays an essential role in the solvent tolerance trait of P. putida S12. The efficient solvent extrusion pump SrpABC (homologous to TtgGHI), the styrene-phenylacetate degradation pathway, and the recently identified toxin-antitoxin SlvTA are encoded within this megaplasmid (18). Unlike P. putida DOT-T1E, P. putida S12 does not encode the toluene degradation pathway within its genome, and thus, its solvent tolerance relies on the gene clusters encoded in pTTS12, as mentioned above (32). However, previous attempts expressing SrpABC in other non-solvent-tolerant bacteria like E. coli were unsuccessful in inciting the same level of solvent tolerance as with P. putida. This may indicate that P. putida S12 is intrinsically solvent tolerant to begin with (33, 34). Hence, in this paper, we further scrutinized this putative intrinsic solvent tolerance in P. putida S12 by using adaptive laboratory evolution (ALE).

Upon curing of megaplasmid pTTS12, the solvent tolerance of P. putida S12 was significantly reduced. After 4 or 5 adaptation cycles to increasing toluene concentrations, the solvent tolerance trait of plasmid-cured P. putida S12 could be restored. Relatively brief adaptation to alternating cycles of LB medium in the presence or absence of toluene can restore solvent tolerance to elevated concentrations of toluene due to the stringent selection pressure elicited by this experimental setup. However, we also observed a severe reduction in growth parameters of the resulting ALE-derived strains grown in the absence of toluene in comparison to wild-type P. putida S12 undergoing the same adaptation cycles to toluene.

During the growth phase in fresh LB medium without toluene addition, induction of solvent tolerance genes is switched off again. The mutants exhibiting constitutive upregulation of the important genes were again positively selected at the next growth phase, with a higher concentration of toluene added to the medium, compared to the cells that require induction to activate their solvent tolerance mechanisms. In addition, the growth phase in fresh LB medium was also important for creating more generations of mutants before a higher toluene concentration was added in the next phase. Indeed, this is not a conventional ALE setup with sustained selective pressure but rather a sequential batch cultivation setup. A similar setup has been successfully implemented in the past to engineer the utilization of mixed carbon sources in Saccharomyces cerevisiae (35).

### Upregulation of a solvent efflux pump is compensated for by downregulation of other membrane proteins.

RNA sequencing revealed constitutive differential changes in gene expression in ALE-derived strains caused by the observed mutations. Truncation of ArpR caused a moderate upregulation of the ArpBC locus, confirming the promiscuous function of the ArpABC efflux pump as an antibiotic pump and a solvent pump, as previously described (36). However, other RND efflux pumps were generally downregulated in ALE-derived strains. Indeed, it has been described that expression of a combination of different efflux pumps can be toxic to bacteria (37). While there may be multifactorial causes of efflux pump toxicity, including membrane composition changes and insertion machinery overload (37–39), we propose that ArpABC activation for solvent extrusion caused increased proton influx. In ALE-derived strains, F_o_F_1_ ATP synthase subunits, flagella, and other H^+^ influx-dependent membrane transporters were severely downregulated following the moderate upregulation of the ArpBC locus. Downregulation of F_o_F_1_ ATP synthase subunits may contribute to the observed fitness reduction and, at the same time, be required as a strategy in ALE-derived strains to overcome efflux pump toxicity in supporting the immense effort of solvent extrusion.

### Truncation of putative regulator Afr in ALE-derived strains reduces expression of membrane proteins.

Indel mutations were observed in a hitherto uncharacterized AraC family transcriptional regulator (Afr), causing it to be truncated in the ALE-derived strains. In P. putida KT2440, a homolog of Afr encoded by PP1395 (100% identity, 100% coverage) was found to be responsible for a decrease in glycerol uptake (40), while in P. aeruginosa PA14, an Afr homolog (63% identity, 88% coverage) encoded by PA14_38040 (PA2074 in strain PAO1) was reported to regulate the expression of RND efflux pump MexEF-OprN (41). Further characterization of Afr is under way. Since the above-mentioned homologs of Afr suggest a role in the regulation of transporters, truncation/deletion of Afr may contribute to the maintenance of proton motive force and membrane composition in ALE-derived strains.

### Mutations in the *gacS* and *gacA* loci as a common strategy for swift phenotypic switching in pseudomonads.

Truncation of the GacS protein and an SNP at the *gacA* locus in ALE-derived strains resulted in the observed upregulation of its target, the *rsmA* locus. Alginate biosynthesis genes, the main polysaccharide constituting *Pseudomonas* biofilm (24), were constitutively downregulated in ALE-derived strains. Indeed, we observed reduced biofilm formation in ALE-derived strains, which could be reversed when the mutation in the *gacS* locus was complemented with wild-type sequence. In P. aeruginosa, biofilm dispersion can be triggered by carbon starvation and involves a proton motive force-dependent step(s) (42). During solvent stress, efficient carbon catabolism and energy production are essential for the extrusion of solvent and the survival of P. putida S12; therefore, biofilm formation causing carbon starvation and oxygen depletion is disadvantageous.

In the reverse engineering strain RE2, the deletion of the *gacS* locus resulted in a significant improvement in growth parameters in the presence and absence of toluene. This mutation may have been selected for to compensate for the mutations that severely affect the growth of ALE-derived strains, e.g., the mutations at F_o_F_1_ ATP synthase loci. A similar observation was also reported in previous studies; e.g., the loss-of-function mutation at the *gacS* and *gacA* loci increased the fitness of plasmid-carrying bacterial strains (43) and improved growth characteristics and efficient root colonization (44, 45). The GacS/GacA two-component system may have a pleiotropic effect, since this system regulates a large number of genes as a response to environmental stimuli. Additionally, the *gacA* and *gacS* loci may constitute commonly mutated loci with an elevated mutation rate to allow for a swift phenotypic switching in response to environmental dynamics (43–45).

### Summary.

In summary, ALE presents a powerful combination of mutation selection and construction of beneficial genetic variation in many different genes and regulatory regions in parallel (46, 47), for the restoration of solvent tolerance in plasmid-cured P. putida S12. Through ALE, we gained insight into intrinsically promoting the solvent tolerance of P. putida S12. The high metabolic flexibility of P. putida S12, e.g., the ability to maintain proton motive force and membrane stabilization, indeed proved essential to incite solvent tolerance with the availability of a solvent extrusion pump. This may very well be under the control of *gacA* and *gacS* loci and may involve the putative regulator Afr. Further characterization of the efficiency of solvent extrusion pumps and their impact and demand on proton motive force is required for the application of solvent-tolerant strains, especially in the bioproduction of high-value chemicals and biofuels.

## MATERIALS AND METHODS

### Strains and culture conditions.

The strains and plasmids used in this paper are listed in Table 3. P. putida strains were grown in lysogeny broth (LB) medium at 30°C with shaking at 200 rpm. E. coli strains were cultivated in LB medium at 37°C with shaking at 250 rpm. For solid cultivation, 1.5% (wt/vol) agar was added to LB medium. When required, gentamicin (25 mg liter^−1^), ampicillin (100 mg liter^−1^), kanamycin (50 mg liter^−1^), and streptomycin (50 mg liter^−1^) were added to the media. Hartman’s minimal medium (13) was supplemented with 2 mg MgSO_4_, and 0.2% (wt/vol) citrate, 0.4% (wt/vol) glycerol, or 0.2% (wt/vol) glucose was added as the sole carbon source when necessary. Growth parameters were measured in a 96-well plate using a Tecan Spark 10M instrument and calculated using growthcurver R package v.0.3.0 (48). The maximum growth rate (μ_max_) was calculated as the highest growth rate when there were no restrictions imposed on the total population size (*t* = 2 to 5 h). The maximum OD_600_ (maxOD) was defined as the OD_600_ measurement after the stationary phase was reached (*t* ≈ 10 h).

**TABLE 3:** Strains and plasmids used in this study

Strain(s) or plasmid	Characteristics	Reference or source
Strains
P. putida S12	Wild-type P. putida S12 (ATCC 700801), harboring megaplasmid pTTS12, solvent-tolerant strain	54
P. putida S12d (S12-06, S12-10, S12-22)	P. putida S12 ΔpTTS12, non-solvent-tolerant strains	19
P. putida S12e (S12-06e30, S12-10e35, S12-22e30)	ALE-derived P. putida S12 ΔpTTS12, solvent-tolerant strain	This paper
P. putida S12e30	ALE-derived wild-type P. putida S12 (control strain)	This paper
P. putida S12d Δ*arpR*	P. putida S12 ΔpTTS12 ΔRPPX_14650	This paper
P. putida S12d Δ*afr*	P. putida S12 ΔpTTS12 ΔRPPX_14685	This paper
P. putida S12d Δ*gacA*	P. putida S12 ΔpTTS12 ΔRPPX_00635	This paper
P. putida S12d Δ*gacS*	P. putida S12 ΔpTTS12 ΔRPPX_15700	This paper
P. putida S12e *arpR*-wt	ALE-derived P. putida S12 ΔpTTS12 RPPX_14650-wt	This paper
P. putida S12e *afr*-wt	ALE-derived P. putida S12 ΔpTTS12 RPPX_14685-wt	This paper
P. putida S12e *gacS*-wt	ALE-derived P. putida S12 ΔpTTS12 RPPX_15700-wt	This paper
P. putida S12e *rpoB*′-wt	ALE-derived P. putida S12 ΔpTTS12 RPPX_06985-wt	This paper
P. putida S12e ATP-wt	ALE-derived P. putida S12 ΔpTTS12 RPPX_09480-09510-wt	This paper
P. putida S12 Δ*lapA*	P. putida S12 ΔRPPX_08475	This paper
P. putida S12 Δ*flg*	P. putida S12 ΔRPPX_02040-02125	This paper
P. putida S12-RE1	P. putida S12-10 (ΔpTTS12) Δ*arpR*	This paper
P. putida S12-RE2	P. putida S12-10 (ΔpTTS12) Δ*arpR* Δ*gacS*	This paper
P. putida S12-RE3	P. putida S12-10 (ΔpTTS12) Δ*arpR* Δ*gacS atpα* (R165C)	This paper
P. putida S12-RE4	P. putida S12-10 (ΔpTTS12) Δ*arpR* Δ*gacS atpα* (R165C) Δ*afr*	This paper
P. putida S12-RE5	P. putida S12-10 (ΔpTTS12) Δ*arpR* Δ*gac; atpα* (R165C) Δ*afr rpoB′* (D622G)	This paper
E. coli WM3064	*thrB1004 pro thi rpsL hsdS lacZ*ΔM15 RP4-1360 Δ(*araBAD*)567 Δ*dapA1341*::[erm pir]	William Metcalf
Plasmids
pEMG	Km^r^ Ap^r^ *ori* R6K *lacZ*α MCS flanked by two I-SceI sites	49
pEMG-Δ*arpR*	pEMG plasmid for constructing P. putida S12d Δ*afr*	This paper
pEMG-Δ*afr*	pEMG plasmid for constructing P. putida S12d Δ*afr*	This paper
pEMG-Δ*gacA*	pEMG plasmid for constructing P. putida S12d Δ*gacA*	This paper
pEMG-Δ*gacS*	pEMG plasmid for constructing P. putida S12d Δ*gacS*	This paper
pEMG-Δ*lapA*	pEMG plasmid for constructing P. putida S12 Δ*lapA*	This paper
pEMG-Δ*flg*	pEMG plasmid for constructing P. putida S12 Δ*flg*	This paper
pEMG-c-*arpR*	pEMG plasmid for constructing P. putida S12e *arpR*-wt	This paper
pEMG-c-*afr*	pEMG plasmid for constructing P. putida S12e *afr*-wt	This paper
pEMG-c-*gacS*	pEMG plasmid for constructing P. putida S12e *gacS*-wt	This paper
pEMG-c-*rpoB′*	pEMG plasmid for constructing P. putida S12e *rpoB′*-wt	This paper
pEMG-c-ATP	pEMG plasmid for constructing P. putida S12e ATP-wt	This paper
pSW-2	Gm^r^ *ori* RK2 *xylS* Pm → I-SceI	49
p421-cas9	Cas9 and tracrRNA; oriV RK2; Sm^r^/Sp^r^	51
p658-ssr	*xylS* ‐Pm → ssr oriV RSF1010; Gm^r^	51
p2316	SEVA CRISPR array; oriV pBBR1; Km^r^	50
p2316-ATPsyn	pSEVA2316 derivative containing the ATP synthase spacer	This paper
p2316-rpoB′-622	pSEVA2316 derivative containing the *rpoB′* spacer (substitution of amino acid D622G)	This paper

Solvent tolerance analysis was performed by growing 20 ml of P. putida culture (starting OD_600_ ± 0.1) on LB medium with the addition of toluene (0.15 to 10% [vol/vol]) in 250-ml Boston glass bottles with Mininert valve (Sigma-Aldrich) bottle caps. Cell turbidity (OD_600_) was measured at time points 0, 4, 24, and 48 h to indicate biomass growth. If biomass growth could not be observed, 1 ml of the liquid culture was plated with serial dilution to determine the bacterial cell survival by counting the CFU. Strain survival in the presence of a high toluene concentration was observed by counting the CFU of 1 ml culture after the addition of 10% (vol/vol) toluene (incubated at 30°C for 30 min) and dividing that number by the CFU of 1 ml starting culture. The average of at least three independent replicates represented the survival frequency of the strain.

### Adaptive laboratory evolution.

P. putida strains were grown overnight on LB medium at 30°C with shaking at 200 rpm. Starting cultures were diluted 100 times with LB medium (starting OD_600_ ± 0.05), and 20-ml volumes of these diluted cultures were placed in Boston bottles. Toluene was added (0.15% [vol/vol]) to the cultures, and the bottles were immediately closed using Mininert bottle caps. These cultures were grown at 30°C with shaking at 200 rpm for approximately 24 to 48 h to allow the strains to reach stationary phase (approximately 7 generations per growth cycle). The toluene-adapted cultures were then diluted 100 times with LB medium and grown overnight at 30°C with shaking at 200 rpm (approximately 10 generations per growth cycle). Stocks were made from this LB culture, and the cycle of toluene adaptation was continued with a higher toluene concentration (0.2% [vol/vol]). This cycle was repeated for up to 8 cycles with the addition of 0.5% (vol/vol) toluene, as shown in Fig. 1.

### PCR and cloning methods.

PCRs were performed using Phusion polymerase (Thermo Fisher) according to the manufacturer’s manual. Oligonucleotides used in this paper (Table 4) were procured from Sigma-Aldrich. PCR products were analyzed by gel electrophoresis on 1% (wt/vol) Tris-borate-EDTA (TBE) agarose containing 5 μg ml^−1^ ethidium bromide (110 V, 0.5× TBE running buffer).

**TABLE 4:** Oligonucleotides used in this study

Oligonucleotide	Sequence	Purpose
Afr_test_F	GAAACGACCATGTAATGC	PCR and Sanger sequencing of mutated region within RPPX_14685
Afr_test_R	GAGGAAAGCCATCATGAC	PCR and Sanger sequencing of mutated region within RPPX_14685
ArpR_test_F	GTCGAACCAAAGAAGAAG	PCR and Sanger sequencing of mutated region within RPPX_14650
ArpR_test_R	GCTGAAGACTACGCAATC	PCR and Sanger sequencing of mutated region within RPPX_14650
ATP_test_F	GGTTATTCGCTACAACTC	PCR and Sanger sequencing of mutated region in the intergenic region of RPPX_09485-09490
ATP_test_R	GAAGATCAACAGGAAGATC	PCR and Sanger sequencing of mutated region in the intergenic region of RPPX_09485-09490
ATPa_test_F	AAAGGTCCTCTGGGTAAC	PCR and Sanger sequencing of mutated region within RPPX_09510
ATPa_test_R	GACAGCAACATAAACACAG	PCR and Sanger sequencing of mutated region within RPPX_09510
GacA_test_F	ATCTGCGGGCTGATATAG	PCR and Sanger sequencing of mutated region within RPPX_000635
GacA_test_R	GTTGTGCTGATGGATGTG	PCR and Sanger sequencing of mutated region within RPPX_000635
GacS_test_F	CGACAGCTCGATCTCTAC	PCR and Sanger sequencing of mutated region within RPPX_15700
GacS_test_R	CAACTGGAGAGAATTGCC	PCR and Sanger sequencing of mutated region within RPPX_15700
rpoB1_test_45_F	ACTTCAACGCCGCACTTC	PCR and Sanger sequencing of mutated region within RPPX_06985, for the mutation N45S
rpoB1_test_45_R	CTTGAAAGACCTACTGAATTTGC	PCR and Sanger sequencing of mutated region within RPPX_06985, for the mutation N45S
rpoB1_test_410_F	TTACCTTCGATCAGTACGG	PCR and Sanger sequencing of mutated region within RPPX_06985, for the mutation D622G
rpoB1_test_410_R	GGTAAGCGTGTTGACTACTCC	PCR and Sanger sequencing of mutated region within RPPX_06985, for the mutation D622G
rpoB1_test_622_F	GTACTGGCTCTCGATTTC	PCR and Sanger sequencing of mutated region within RPPX_06985, for the mutation D410E
rpoB1_test_622_R	TTGTACTGGGTCTGTACTACAT	PCR and Sanger sequencing of mutated region within RPPX_06985, for the mutation D410E
Flg_test_F	CATACATTTCGCGGTAGAC	PCR and Sanger sequencing of the knocked-out flagellar gene cluster
Flg_test_F	AGAATAAGCAGTACCTGGTTTC	PCR and Sanger sequencing of the knocked-out flagellar gene cluster
KO_TS1_ArpR_F	CGGGCGAATTCTCTTCTTTCTTACAGCCACT	ΔRPPX_14650
KO_TS1_ArpR_R	AACAGATCGACACTGTCAGCTTGTAGAAGGCCCTTTC	ΔRPPX_14650
KO_TS2_ArpR_F	GAAAGGGCCTTCTACAAGCTGACAGTGTCGATCTGTT	ΔRPPX_14650
KO_TS2_ArpR_R	CGGACTCTAGACTACAAACCACAGATCCTG	ΔRPPX_14650
KO_TS1_Afr_F	CCTCAGGATCCCTCAGTCGAAGGTATGCAAGTCCA	ΔRPPX_14685
KO_TS1_Afr_R	TTCTTCACCTTTATGAATTCCCATGATCATGATGGCTTTCCTCTGCTGACCG	ΔRPPX_14685
KO_TS2_Afr_F	CGCACGGCATGGATGAACTCTACAAATAAAGCTCAACATTCGCCTGCACCG	ΔRPPX_14685
KO_TS2_Afr_R	GGCGATCTAGACCAACCTGTGTATACCGGCAACCT	ΔRPPX_14685
KO_TS1_GacA_F	TAGTAGCTATCTTCGCACTG	ΔRPPX_00635
KO_TS1_GacA_R	CTCATGTCCCAAGTCTTT	ΔRPPX_00635
KO_TS2_GacA_F	ACCACTAAGACCCTAATCAA	ΔRPPX_00635
KO_TS2_GacA_R	CATAGTGAATCCATACGTTTAC	ΔRPPX_00635
KO_TS1_GacS_F	GAGTTCGTCATTGATGAAG	ΔRPPX_15700
KO_TS1_GacS_R	CTGACATGCTGAGTATGCT	ΔRPPX_15700
KO_TS2_GacS_F	ATCCTTGAGCTGATTGAC	ΔRPPX_15700
KO_TS2_GacS_R	GGTAGCTATTTCACCTGG	ΔRPPX_15700
KO_TS1_lapA_F	AGATTGAATTCAAGTACAACCATATAACTGTCC	Knocking-out *lapA* (adhesin), negative control for biofilm assay
KO_TS1_lapA_R	GTGGCGTAATCGTTTATAATCATCATCCACAACAAG	Knocking-out *lapA* (adhesin), negative control for biofilm assay
KO_TS2_lapA_F	CTTGTTGTGGATGATGATTATAAACGATTACGCCAC	Knocking-out *lapA* (adhesin), negative control for biofilm assay
KO_TS2_lapA_R	GGGCATCTAGAGTGTACTTATCGAAGGTGAC	Knocking-out *lapA* (adhesin), negative control for biofilm assay
KO_TS1_flg_F	GGCAGGGATCCGGAAGAAATTTCACCTAAAAGC	Knocking-out flagellar gene cluster, negative control for swimming motility assay
KO_TS1_flg_R	CTATACCTTGCTCAACGAAATTGAAGAGATGATGCAGATCAATC	Knocking-out flagellar gene cluster, negative control for swimming motility assay
KO_TS2_flg_F	GATTGATCTGCATCATCTCTTCAATTTCGTTGAGCAAGGTATAG	Knocking-out flagellar gene cluster, negative control for swimming motility assay
KO_TS2_flg_R	CGTGTTCTAGAGGAATGTGCTGATCTATTTCTC	Knocking-out flagellar gene cluster, negative control for swimming motility assay
c_Afr_F	GCAATGAATTCATCTGTGCAAAAACCTGTA	Complementation of RPPX_14685 to the wild-type sequence in the evolved strains
c_Afr_R	AGGGTTCTAGAACAATCTCCTTGAAGCAG	Complementation of RPPX_14685 to the wild-type sequence in the evolved strains
c_ArpR_F	CCTTCGAATTCCGAAGAGCGACCGATACGG	Complementation of RPPX_14650 to the wild-type sequence in the evolved strains
c_ArpR_R	TTCTTTCTAGACTTCCAGTCCATTTCGCTGACC	Complementation of RPPX_14650 to the wild-type sequence in the evolved strains
c_ATPa_F	CGAAAGAATTCGAAGCATTGAAATCTTGAG	Complementation of RPPX_09510 to the wild-type sequence in the evolved strains
c_ATPa_R	TGATGTCTAGAATCTTACTGCGAATCTCTTT	Complementation of RPPX_09510 to the wild-type sequence in the evolved strains
c_GacA_F	GCGCTGAATTCAAAGACTTGGGACATGAG	Complementation of RPPX_00635 to the wild-type sequence in the evolved strains
c_GacA_R	GAGGTGGATCCTTGATTAGGGTCTTAGTGGT	Complementation of RPPX_00635 to the wild-type sequence in the evolved strains
c_GacS_F	CAGCCGAATTCAAGCTTTCCTGATCGTAG	Complementation of RPPX_15700 to the wild-type sequence in the evolved strains
c_GacS_R	GGCAATCTAGAATAACACGTACTAAAGAGATGC	Complementation of RPPX_15700 to the wild-type sequence in the evolved strains
c_ATP_F	GCACTGGATCCACAACTTAAGGAATCCGTAT	Complementation of RPPX_09485-09490 to the wild-type sequence in the evolved strains
c_ATP_R	GTCAGTCTAGACAAGTGGTGCTGGATATAG	Complementation of RPPX_09485-09490 to the wild-type sequence in the evolved strains
c_rpoB1_F	CCAAAGGATCCGGTACAAAAGTCTAAAGAGGAT	Complementation of RPPX_06985 to the wild-type sequence in the evolved strains
c_rpoB1_R	AGGAGTCTAGACCTTGAAAGACCTACTGAAT	Complementation of RPPX_06985 to the wild-type sequence in the evolved strains
cr-rpoBWT-622-1-S	AAACAGTAAGCGAAACCGGTGTACATCAGCTGGTG	Spacer fragment for introducing D622G point mutation at RPPX_06985
cr-rpoBWT-622-1-AS	AAAACACCAGCTGATGTACACCGGTTTCGCTTACT	Spacer fragment for introducing D622G point mutation at RPPX_06985
RpoB-622e-1	GAAATGGTCGAGTAAGCGAAACCGGTGTACATCAGCTGGCCGGCGAAGATAACGGTCTCTTTCAGACCAACCACGCGGTAG	Repair fragment for introducing D622G point mutation at RPPX_06985
cr-ATPWT-1-S	AAACATCTGACGGTCGCCAATGATCAGCTCACGCG	Spacer fragment for introducing R165C point mutation at RPPX_09510
cr-ATPWT-1-AS	AAAACGCGTGAGCTGATCATTGGCGACCGTCAGAT	Spacer fragment for introducing R165C point mutation at RPPX_09510
ATP-09510e-1	GTCTTGCCGATCTGACGGTCGCCAATGATCAGCTCACACTGGCCACGGCCGACAGGGATCATGGCGTCGACGGATTTGTAACCAG	Repair fragment for introducing R165C point mutation at RPPX_09510

Deletion of *arpR*, *gacA*, *gacS*, and *afr* genes and restoration of mutations in ALE-derived strains were performed using homologous recombination between free-ended DNA sequences that are generated by cleavage on unique I-SceI sites (49). Two homologous recombination fragments (TS-1 and TS-2) were obtained by performing PCR using the oligonucleotides listed in Table 4. Reverse engineering of the point mutations at ATP synthase subunit α (RPPX_09510) and RNA polymerase subunit β′ (RPPX_06985) loci were performed using the CRISPR-cas9 enhanced single-stranded DNA (ssDNA) recombineering method (50, 51). Spacers and ssDNA repair fragments were created using the oligonucleotides listed in Table 4. All of the obtained plasmid constructs, deletion, and restoration of the selected genes were verified by Sanger sequencing (Macrogen BV, Amsterdam, The Netherlands).

### Whole-genome sequencing of plasmid-cured and ALE-derived strains.

For whole-genome sequencing, DNA was extracted by phenol-chloroform extraction, followed by column cleanup using a NucleoSpin DNA Plant II kit (Macherey-Nagel). Clustering and DNA sequencing of wild-type, plasmid-cured, and ALE-derived P. putida S12 strains were performed using Illumina cBot and HiSeq 4000 systems (GenomeScan BV, The Netherlands). Image analysis, base calling, and quality checking were performed with the Illumina data analysis pipeline RTA v.2.7.7 and Bcl2fastq v.2.17. Sequencing reads were assembled according to the existing complete genome sequence (GenBank accession no. CP009974 and CP009975) in Geneious software (18). Variant calling was performed by aligning the reads from the ALE-derived strains to their corresponding parental strains.

### RNA sequencing of plasmid-cured and ALE-derived strains.

Wild-type, plasmid-cured, and ALE-derived P. putida S12 cultures were grown from overnight culture (100 times diluted) in 20 ml LB medium for 2 h (30°C, 200 rpm) with and without the addition of 0.1% (vol/vol) toluene to bacterial cell cultures. RNA was extracted using TRIzol reagent (Invitrogen) according to the manufacturer’s manual. The obtained RNA samples were cleaned up using a NucleoSpin RNA plant and fungi kit (Macherey-Nagel). To enrich the samples, rRNAs were depleted using an Illumina Ribo-Zero rRNA depletion kit prior to the library preparation. Illumina RNA libraries were prepared for sequencing using standard Illumina protocols, and paired-end sequence reads were generated using the Illumina MiSeq system (BaseClear BV, The Netherlands). Initial quality assessment was based on data passing Illumina Chastity filtering. Subsequently, reads containing the PhiX control signal were removed using an in-house filtering protocol. In addition, reads containing (partial) adapters were clipped (up to a minimum read length of 50 bp). The second quality assessment was based on the remaining reads using the FASTQC quality control tool, version 0.11.5. Tophat2 version 2.1.1 aligned RNA-seq reads to a reference genome (GenBank accession no. CP009974 and CP009975) using the ultrahigh-throughput short read aligner Bowtie version 2.2.6 (52, 53). Cufflink was used to test for differential expression and regulation in RNA-seq samples. Cuffdiff then estimated the relative abundances of these transcripts.

### Microtiter dish biofilm formation assay.

To quantify biofilm formation, a crystal violet-based assay on a 96-well plate was performed as described by O’Toole (25). Overnight cultures of P. putida S12 (100 times diluted) were grown in a flat-bottomed 96-well microtiter plate with 100 μl LB medium (30°C) for 6 h without shaking. After 6 h, the OD_600_ was measured using a Tecan Spark 10M (Tecan) instrument to ensure the growth of P. putida S12. Liquid cultures were removed from the 96-well microtiter plate and then washed two times with water. Crystal violet solution (0.1% [vol/vol]), 125 μl, was added to each well, followed by 10 to 15 min of incubation. After incubation, the crystal violet solution was removed and the wells were washed with water to remove the excess crystal violet. The microtiter plate was then turned upside down and dried. Acetic acid solution (30% [vol/vol]), 125 μl, was added to solubilized biofilm, stained with crystal violet, and incubated for 10 to 15 min. The absorbance at 550 nm was measured using a Tecan Spark 10M (Tecan) instrument to represent biofilm formation, with acetic acid solution as a blank.

### Swimming motility assay.

As a starting culture, P. putida S12 strains were streaked and grown on LB agar overnight (30°C). Single colonies were picked and stab inoculated onto low-viscosity LB agar (0.3% [wt/vol] agar). This agar was incubated cap side up for 24 h at 30°C. Radial growth of P. putida S12 on low-viscosity agar was measured with three replicates to represent swimming motility.

### Data availability.

Whole-genome sequencing data for the wild-type, plasmid-cured genotype, and ALE-derived P. putida S12 strains have been submitted to the SRA database under accession number PRJNA602416. Data sets generated from RNA-seq experiments have been submitted to the GEO database under accession number GSE144045.

## Supplementary Material

Supplemental file 1
