# Supplementary material for: Adaptive Laboratory Evolution Restores Solvent Tolerance in Plasmid-Cured Pseudomonas putida S12: a Molecular Analysis
Source: Appl Environ Microbiol. 2021 Apr 13;87(9):e00041-21. doi: 10.1128/AEM.00041-21 (PMC8091024; doi:10.1128/AEM.00041-21)

Supplemental Materials

Adaptive laboratory evolution restores solvent tolerance in plasmid-cured *Pseudomonas putida* S12; a molecular analysis

Hadiastri Kusumawardhani, Benjamin Furtwängler, Matthijs Blommestijn, Adelė Kaltenytė, Jaap van der Poel, Jan Kolk, Rohola Hosseini, Johannes H. de Winde*

Institute of Biology Leiden, Leiden University, The Netherlands

*Correspondence: j.h.de.winde@biology.leidenuniv.nl

Table S1. Bacground mutations on plasmid-cured P. putida S12 strains (due to the Mitomycin C treatment)

Strain	Minimum	Maximum	Length	Amino Acid Change	CDS Position	Change	Codon Change	Coverage	Polymorphism Type	Protein Effect	Variant Frequency	Variant P-Value (approximate)
S12.6	1,256,943	1,256,943	1			G -> C		295	SNP (transversion)		99.7%	1.9E-1086
S12.22	2,812,212	2,812,212	1			A -> G		188	SNP (transition)		34.0%	2.1E-93
S12.6	3,316,341	3,316,341	1	T -> P	157	T -> G	ACC -> CCC	267	SNP (transversion)	Substitution	99.3%	1.0E-1007
S12.10	3,316,341	3,316,341	1	T -> P	157	T -> G	ACC -> CCC	368	SNP (transversion)	Substitution	99.7%	4.6E-1393
S12.22	3,316,341	3,316,341	1	T -> P	157	T -> G	ACC -> CCC	238	SNP (transversion)	Substitution	100.0%	4.0E-905

Table S2. Identified Mutations in ALE-derived Strains

indicates common mutated loci shared among the ALE-derived strains (presented in Figure 2)

Strain	Parent Strain	Name	Minimum	Maximum	Length	Amino Acid Change	CDS Position	Change	Codon Change	Coverage	Polymorphism Type	Protein Effect	Variant Frequency	Variant P-Value (approximate)
S12e30_chr	S12_chr	A	3479624	3479624	1	X -> E	788	M -> A	GMG -> GAG	245	SNP	Substitution	100.0%	1.0E-98
S12e30_pTTS12	S12_pTTS12	G	392630	392630	1	E -> G	179	A -> G	GAG -> GGG	211	SNP (transition)	Substitution	99.5%	1.0E-819
S12e30_pTTS12	S12_pTTS12	TT	432130	432131	2	L -> Q	383	CA -> TT	CTG -> CAA	232 -> 233	Substitution	Substitution	94.4%	3.5E-728
S12-06e30	S12-06	A	7117	7117	1	S -> N	71	G -> A	AGC -> AAC	286	SNP (transition)	Substitution	100.0%	4.0E-973
S12-06e30	S12-06	A	552325	552325	1		1,296	G -> A	CCG -> CCA	289	SNP (transition)	None	99.3%	4.0E-919
S12-06e30	S12-06	A	652139	652139	1		219	G -> A	CTG -> CTA	293	SNP (transition)	None	100.0%	7.9E-1085
S12-06e30	S12-06	A	1088368	1088368	1	A -> T	613	G -> A	GCC -> ACC	332	SNP (transition)	Substitution	98.2%	4.1E-1171
S12-06e30	S12-06	C	1256943	1256943	1			G -> C		324	SNP (transversion)		100.0%	4.0E-1167
S12-06e30	S12-06	A	1567277	1567277	1		1,359	G -> A	TCG -> TCA	314	SNP (transition)	None	99.4%	6.3E-1124
S12-06e30	S12-06	C	1595339	1595339	1	D -> G	1,865	T -> C	GAC -> GGC	290	SNP (transition)	Substitution	100.0%	1.0E-1102
S12-06e30	S12-06	A	1877824	1877824	1	R -> Q	596	G -> A	CGG -> CAG	350	SNP (transition)	Substitution	98.3%	3.6E-1302
S12-06e30	S12-06	A	2024784	2024784	1			G -> A		325	SNP (transition)		99.1%	9.6E-1222
S12-06e30	S12-06	A	2065373	2065373	1		246	G -> A	GCC -> GCT	327	SNP (transition)	None	99.7%	1.6E-1239
S12-06e30	S12-06	T	2153472	2153472	1	R -> C	493	C -> T	CGT -> TGT	355	SNP (transition)	Substitution	100.0%	3.2E-1314
S12-06e30	S12-06	T	2362204	2362204	1		264	C -> T	CAG -> CAA	385	SNP (transition)	None	99.7%	1.0E-1344
S12-06e30	S12-06		2367033	2367033	1			(C)10 -> (C)9		298	Deletion (tandem repeat)		96.3%	1.6E-425
S12-06e30	S12-06	G	2367792	2367792	1			A -> G		301	SNP (transition)		99.7%	1.0E-1110
S12-06e30	S12-06	A	2847391	2847391	1	A -> V	206	G -> A	GCC -> GTC	314	SNP (transition)	Substitution	99.4%	1.0E-1092
S12-06e30	S12-06	T	3098332	3098332	1			C -> T		324	SNP (transition)		99.1%	1.6E-1121
S12-06e30	S12-06	G	3303294	3303294	1	V -> A	2,705	A -> G	GTA -> GCA	337	SNP (transition)	Substitution	33.2%	2.0E-323
S12-06e30	S12-06	A	3308004	3308004	1	G -> R	592	G -> A	GGG -> AGG	310	SNP (transition)	Substitution	99.7%	6.3E-1175
S12-06e30	S12-06	G	3316341	3316341	1	T -> P	157	T -> G	ACC -> CCC	316	SNP (transversion)	Substitution	100.0%	6.3E-1170
S12-06e30	S12-06	G	3316345	3316344	0		154	(G)3 -> (G)4		317	Insertion (tandem repeat)	Frame Shift	99.7%	6.3E-1170
S12-06e30	S12-06		3509271	3509271	1		124	(T)5 -> (T)4		294	Deletion (tandem repeat)	Frame Shift	100.0%	4.0E-471
S12-06e30	S12-06		3528741	3528741	1		316	(G)6 -> (G)5		361	Deletion (tandem repeat)	Frame Shift	99.7%	1.2E-608
S12-06e30	S12-06	T	3544782	3544782	1	G -> S	160	C -> T	GGC -> AGC	320	SNP (transition)	Substitution	99.7%	5.0E-1181
S12-06e30	S12-06	G	3549535	3549535	1	D -> G	338	A -> G	GAC -> GGC	333	SNP (transition)	Substitution	99.7%	4.0E-1229
S12-06e30	S12-06	G	3629509	3629509	1	T -> A	1,312	A -> G	ACC -> GCC	312	SNP (transition)	Substitution	99.0%	6.3E-1175
S12-06e30	S12-06	T	3782052	3782052	1		327	C -> T	GAC -> GAT	298	SNP (transition)	None	99.3%	6.5E-1095
S12-06e30	S12-06	A	4258926	4258926	1	G -> D	1,328	G -> A	GGC -> GAC	319	SNP (transition)	Substitution	99.1%	1.0E-1071
S12-06e30	S12-06	G	4402103	4402103	1		522	A -> G	GGA -> GGG	289	SNP (transition)	None	100.0%	5.0E-1070
S12-06e30	S12-06	C	4707728	4707727	0		1,975	(C)5 -> (C)6		296	Insertion (tandem repeat)	Frame Shift	100.0%	6.3E-1096
S12-06e30	S12-06	C	4774024	4774024	1		501	T -> C	GCA -> GCG	370	SNP (transition)	None	99.5%	4.3E-1431
S12-06e30	S12-06	C	5093860	5093860	1	D -> G	137	T -> C	GAC -> GGC	314	SNP (transition)	Substitution	99.7%	1.6E-1127
S12-06e30	S12-06	C	5316112	5316111	0		801	(C)6 -> (C)7		293	Insertion (tandem repeat)	Frame Shift	99.7%	6.3E-1052
S12-06e30	S12-06		5609474	5609474	1		258	(G)3 -> (G)2		313	Deletion (tandem repeat)	Frame Shift	99.7%	3.7E-527
S12-06e30	S12-06	A	5679391	5679391	1		2,481	G -> A	GGG -> GGA	301	SNP (transition)	None	99.0%	1.8E-1041
S12-06e30	S12-06	G	5711295	5711294	0			(G)12 -> (G)13		188	Insertion (tandem repeat)		87.8%	1.7E-412
S12-10e35	S12-10	T	179236	179236	1		186	C -> T	GTG -> GTA	218	SNP (transition)	None	100.0%	2.5E-807
S12-10e35	S12-10	A	201510	201510	1	G -> S	1,459	G -> A	GGC -> AGC	267	SNP (transition)	Substitution	99.3%	3.2E-981
S12-10e35	S12-10		266475	266475	1		198	(C)5 -> (C)4		251	Deletion (tandem repeat)	Frame Shift	100.0%	3.2E-377
S12-10e35	S12-10	T	378163	378163	1	T -> I	404	C -> T	ACC -> ATC	225	SNP (transition)	Substitution	92.0%	1.1E-719
S12-10e35	S12-10		530690	530690	1			(C)6 -> (C)5		236	Deletion (tandem repeat)		97.9%	3.7E-352
S12-10e35	S12-10	C	603320	603319	0		915	(C)7 -> (C)8		255	Insertion (tandem repeat)	Frame Shift	98.4%	3.1E-902
S12-10e35	S12-10	C	746622	746622	1		879	T -> C	GCA -> GCG	233	SNP (transition)	None	99.6%	2.9E-880
S12-10e35	S12-10	C	776207	776206	0		1,308	(C)6 -> (C)7		294	Insertion (tandem repeat)	Frame Shift	99.0%	4.8E-1076
S12-10e35	S12-10	T	1032321	1032321	1	T -> I	1,199	C -> T	ACC -> ATC	230	SNP (transition)	Substitution	99.1%	2.1E-843
S12-10e35	S12-10	C	1057315	1057315	1	T -> A	1,000	T -> C	ACC -> GCC	240	SNP (transition)	Substitution	99.2%	1.2E-853
S12-10e35	S12-10	G	1171289	1171289	1	D -> G	728	A -> G	GAC -> GGC	278	SNP (transition)	Substitution	95.7%	2.2E-964
S12-10e35	S12-10		1250094	1250094	1		661	(C)9 -> (C)8		216	Deletion (tandem repeat)	Frame Shift	99.5%	2.7E-298
S12-10e35	S12-10	T	1481188	1481188	1	D -> N	76	C -> T	GAC -> AAC	258	SNP (transition)	Substitution	93.4%	3.8E-821
S12-10e35	S12-10	A	1530147	1530147	1		102	G -> A	GGC -> GGT	270	SNP (transition)	None	98.5%	2.7E-930
S12-10e35	S12-10	G	1567255	1567255	1	K -> R	1,337	A -> G	AAG -> AGG	242	SNP (transition)	Substitution	99.6%	1.9E-890
S12-10e35	S12-10	C	1575460	1575460	1	N -> S	587	T -> C	AAC -> AGC	214	SNP (transition)	Substitution	100.0%	6.3E-814
S12-10e35	S12-10	C	1597070	1597070	1	N -> S	134	T -> C	AAC -> AGC	249	SNP (transition)	Substitution	100.0%	6.3E-947
S12-10e35	S12-10	A	1620647	1620647	1	G -> S	157	G -> A	GGC -> AGC	274	SNP (transition)	Substitution	85.8%	8.5E-824
S12-10e35	S12-10	T	1646051	1646051	1	G -> S	361	C -> T	GGC -> AGC	256	SNP (transition)	Substitution	97.7%	4.0E-893
S12-10e35	S12-10	A	1663844	1663844	1			G -> A		266	SNP (transition)		98.9%	7.9E-919
S12-10e35	S12-10	A	1673325	1673325	1	A -> T	52	G -> A	GCC -> ACC	257	SNP (transition)	Substitution	99.6%	6.3E-948
S12-10e35	S12-10	A	1689175	1689175	1		66	G -> A	GCC -> GCT	262	SNP (transition)	None	99.2%	1.0E-962
S12-10e35	S12-10	A	1692228	1692228	1		600	G -> A	GCC -> GCT	266	SNP (transition)	None	99.6%	3.4E-979
S12-10e35	S12-10	A	1695381	1695381	1		478	G -> A	CTG -> TTG	275	SNP (transition)	None	94.5%	2.5E-916
S12-10e35	S12-10	T	1711074	1711074	1		636	C -> T	CTC -> CTT	296	SNP (transition)	None	99.7%	3.2E-1092
S12-10e35	S12-10	C	2099811	2099811	1	Q -> R	755	T -> C	CAG -> CGG	258	SNP (transition)	Substitution	99.2%	1.0E-920
S12-10e35	S12-10		2149786	2149786	1		622	(G)5 -> (G)4		282	Deletion (tandem repeat)	Frame Shift	100.0%	4.0E-480
S12-10e35	S12-10	C	2150083	2150083	1			T -> C		307	SNP (transition)		98.7%	1.4E-1143
S12-10e35	S12-10		2367033	2367033	1			(C)10 -> (C)9		230	Deletion (tandem repeat)		94.8%	7.7E-291
S12-10e35	S12-10	G	2371742	2371742	1	D -> G	1,181	A -> G	GAT -> GGT	259	SNP (transition)	Substitution	99.6%	1.6E-929
S12-10e35	S12-10	T	2424530	2424530	1	G -> D	881	C -> T	GGT -> GAT	256	SNP (transition)	Substitution	98.8%	4.1E-935
S12-10e35	S12-10	T	2533014	2533014	1	G -> D	977	C -> T	GGC -> GAC	246	SNP (transition)	Substitution	98.8%	2.9E-847
S12-10e35	S12-10	G	2544029	2544028	0		1,911	(G)8 -> (G)9		248	Insertion (tandem repeat)	Frame Shift	98.0%	2.3E-870
S12-10e35	S12-10	G	2579936	2579935	0		168	(G)9 -> (G)10		269	Insertion (tandem repeat)	Frame Shift	91.8%	8.0E-870
S12-10e35	S12-10	A	2600879	2600879	1	P -> S	589	G -> A	CCG -> TCG	255	SNP (transition)	Substitution	87.8%	4.3E-768
S12-10e35	S12-10	C	2663683	2663682	0		36	(C)6 -> (C)7		257	Insertion (tandem repeat)	Frame Shift	100.0%	3.2E-900
S12-10e35	S12-10	T	2701181	2701181	1	G -> E	746	C -> T	GGG -> GAG	267	SNP (transition)	Substitution	97.8%	1.4E-885

Strain	Parent Strain	Name	Minimum	Maximum	Length	Amino Acid Change	CDS Position	Change	Codon Change	Coverage	Polymorphism Type	Protein Effect	Variant Frequency	Variant P-Value (approximate)
S12-10e35	S12-10	A	2709165	2709165	1	A -> T	565	G -> A	GCC -> ACC	217	SNP (transition)	Substitution	93.1%	2.1E-686
S12-10e35	S12-10	C	2735364	2735363	0		233	(C)3 -> (C)4		253	Insertion (tandem repeat)	Frame Shift	99.2%	1.8E-903
S12-10e35	S12-10	G	2812212	2812212	1			A -> G		216	SNP (transition)		30.1%	4.6E-97
S12-10e35	S12-10	C	2920552	2920552	1	V -> A	986	T -> C	GTG -> GCG	269	SNP (transition)	Substitution	99.3%	2.9E-1014
S12-10e35	S12-10	G	2922216	2922216	1		27	A -> G	CCT -> CCC	268	SNP (transition)	None	91.0%	1.4E-852
S12-10e35	S12-10	T	2966285	2966285	1	A -> T	229	C -> T	GCT -> ACT	285	SNP (transition)	Substitution	92.3%	4.6E-971
S12-10e35	S12-10	T	3056701	3056701	1	G -> D	668	C -> T	GGC -> GAC	267	SNP (transition)	Substitution	97.0%	2.2E-872
S12-10e35	S12-10	G	3097502	3097501	0		984	(G)6 -> (G)7		295	Insertion (tandem repeat)	Frame Shift	100.0%	1.0E-1062
S12-10e35	S12-10	G	3101879	3101878	0		843	(G)6 -> (G)7		257	Insertion (tandem repeat)	Frame Shift	99.6%	2.5E-922
S12-10e35	S12-10	T	3109593	3109593	1			C -> T		225	SNP (transition)		100.0%	1.0E-810
S12-10e35	S12-10		3146925	3146925	1			(G)8 -> (G)7		234	Deletion (tandem repeat)		100.0%	4.0E-375
S12-10e35	S12-10	T	3249350	3249350	1	A -> V	68	C -> T	GCC -> GTC	232	SNP (transition)	Substitution	98.3%	2.4E-797
S12-10e35	S12-10	T	3297187	3297187	1	T -> I	833	C -> T	ACC -> ATC	258	SNP (transition)	Substitution	89.1%	8.2E-794
S12-10e35	S12-10	G	3303300	3303300	1	V -> A	2,699	A -> G	GTG -> GCG	266	SNP (transition)	Substitution	99.2%	5.6E-973
S12-10e35	S12-10	G	3307552	3307551	0		140	(G)6 -> (G)7		261	Insertion (tandem repeat)	Frame Shift	97.3%	1.6E-933
S12-10e35	S12-10	G	3316341	3316341	1	T -> P	157	T -> G	ACC -> CCC	253	SNP (transversion)	Substitution	100.0%	4.0E-962
S12-10e35	S12-10	G	3316345	3316344	0		154	(G)3 -> (G)4		250	Insertion (tandem repeat)	Frame Shift	99.6%	4.0E-897
S12-10e35	S12-10	G	3419216	3419216	1		798	A -> G	CAA -> CAG	263	SNP (transition)	None	99.6%	2.5E-996
S12-10e35	S12-10	G	3452892	3452892	1	Y -> H	1,156	A -> G	TAC -> CAC	253	SNP (transition)	Substitution	99.6%	2.5E-958
S12-10e35	S12-10	A	3479624	3479624	1	X -> E	788	M -> A	GMG -> GAG	222	SNP	Substitution	99.5%	5.6E-65
S12-10e35	S12-10	G	3528742	3528741	0		316	(G)6 -> (G)7		268	Insertion (tandem repeat)	Frame Shift	99.3%	2.5E-958
S12-10e35	S12-10	T	3537413	3537413	1	A -> V	329	C -> T	GCC -> GTC	262	SNP (transition)	Substitution	89.3%	4.2E-856
S12-10e35	S12-10	G	3690651	3690651	1	Y -> C	2,177	A -> G	TAC -> TGC	237	SNP (transition)	Substitution	91.1%	3.0E-794
S12-10e35	S12-10	T	3748119	3748119	1			C -> T		246	SNP (transition)		99.2%	2.5E-830
S12-10e35	S12-10	C	3858978	3858978	1		639	T -> C	CGT -> CGC	242	SNP (transition)	None	100.0%	2.5E-920
S12-10e35	S12-10	G	3865096	3865096	1	D -> A	3,089	T -> G	GAC -> GCC	221	SNP (transversion)	Substitution	99.5%	1.4E-768
S12-10e35	S12-10	G	3946605	3946604	0			(G)7 -> (G)8		214	Insertion (tandem repeat)		95.3%	8.8E-624
S12-10e35	S12-10	G	4176584	4176583	0		474	(G)5 -> (G)6		230	Insertion (tandem repeat)	Frame Shift	95.7%	1.3E-784
S12-10e35	S12-10	T	4221641	4221641	1	R -> C	1,192	C -> T	CGT -> TGT	253	SNP (transition)	Substitution	99.6%	4.0E-933
S12-10e35	S12-10	G	4463943	4463942	0		275	(G)7 -> (G)8		243	Insertion (tandem repeat)	Frame Shift	99.6%	4.0E-896
S12-10e35	S12-10	G	4666198	4666198	1		1,179	A -> G	GGT -> GGC	232	SNP (transition)	None	95.7%	2.5E-809
S12-10e35	S12-10	G	4711920	4711919	0		284	(G)5 -> (G)6		262	Insertion (tandem repeat)	Frame Shift	99.6%	2.5E-940
S12-10e35	S12-10	A	4742974	4742974	1	G -> D	476	G -> A	GGC -> GAC	234	SNP (transition)	Substitution	95.3%	2.3E-810
S12-10e35	S12-10	T	4743032	4743032	1		534	C -> T	CGC -> CGT	220	SNP (transition)	None	33.2%	1.2E-212
S12-10e35	S12-10	G	4836001	4836000	0		1,033	(G)6 -> (G)7		247	Insertion (tandem repeat)	Frame Shift	91.9%	4.2E-824
S12-10e35	S12-10	G	4928278	4928278	1	F -> L	943	A -> G	TTC -> CTC	256	SNP (transition)	Substitution	99.6%	1.0E-969
S12-10e35	S12-10	C	5043280	5043279	0		721	(C)8 -> (C)9		208	Insertion (tandem repeat)	Frame Shift	99.0%	1.6E-783
S12-10e35	S12-10	C	5146750	5146750	1	T -> A	508	T -> C	ACG -> GCG	227	SNP (transition)	Substitution	88.5%	2.2E-734
S12-10e35	S12-10	C	5167012	5167012	1	V -> A	233	T -> C	GTC -> GCC	233	SNP (transition)	Substitution	99.1%	3.4E-832
S12-10e35	S12-10	A	5424887	5424887	1		2,610	G -> A	AGC -> AGT	269	SNP (transition)	None	97.8%	3.3E-918
S12-10e35	S12-10	G	5530225	5530225	1	S -> P	220	A -> G	TCG -> CCG	245	SNP (transition)	Substitution	98.8%	6.4E-891
S12-10e35	S12-10	G	5594662	5594662	1		882	A -> G	GAA -> GAG	219	SNP (transition)	None	99.1%	6.7E-803
S12-10e35	S12-10	C	5602034	5602034	1	L -> P	728	T -> C	CTC -> CCC	225	SNP (transition)	Substitution	99.1%	4.1E-800
S12-10e35	S12-10	A	5610231	5610231	1	E -> K	1,015	G -> A	GAA -> AAA	249	SNP (transition)	Substitution	98.0%	1.0E-851
S12-10e35	S12-10		5711294	5711294	1			(G)12 -> (G)11		136	Deletion (tandem repeat)		96.3%	3.1E-144
S12-22e30	S12-22	A	142041	142041	1	P -> L	269	G -> A	CCC -> CTC	249	SNP (transition)	Substitution	98.8%	6.3E-911
S12-22e30	S12-22	C	1595974	1595974	1	D -> E	1,230	G -> C	GAC -> GAG	310	SNP (transversion)	Substitution	99.7%	6.2E-1142
S12-22e30	S12-22	C	2150602	2150602	1			G -> C		259	SNP (transversion)		100.0%	6.3E-985
S12-22e30	S12-22		3316341	3316341	1		157	-T		253	Deletion	Frame Shift	100.0%	4.0E-456
S12-22e30	S12-22		RPPX_RS14645 - RPPX_RS14650								insertion of mobile element ISS12			

Figure S1. Volcano plot of differential gene expression in wild-type, plasmid-cured, and ALE-derived *P. putida* S12 strains in the presence of 0.1% (vol/vol) toluene growing on LB.

Blue area indicates the significantly upregulated genes and beige area indicates the significantly downregulated genes (cut-off: Log_2 Fold Change ≥ 1 for up-regulated genes or ≤ 1 for down-regulated genes and FDR-adjusted p-value ≤ 0.05). The black dots represent the significantly up-/down-regulated genes.

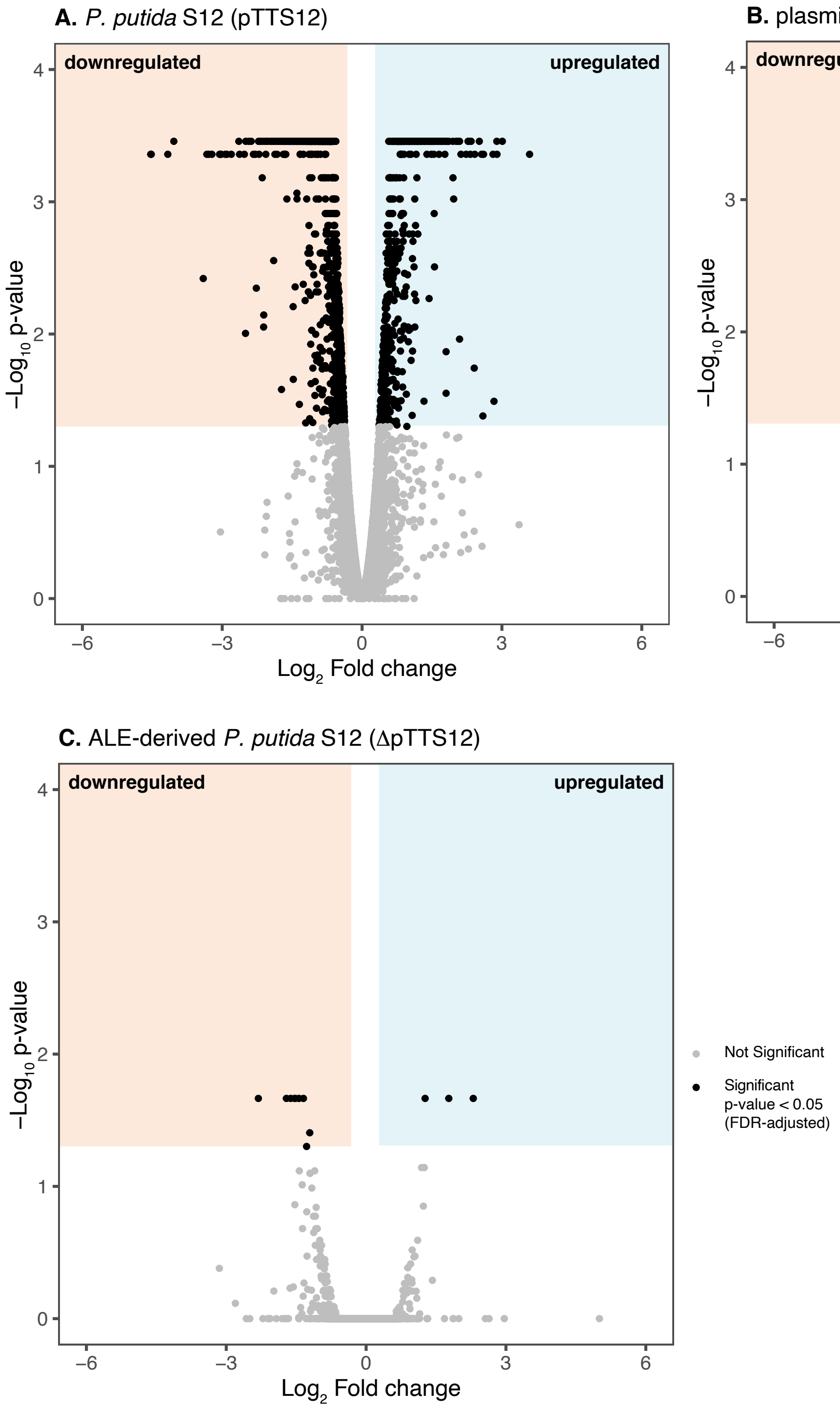

Supplement: Supplemental file 1 [file AEM.00041-21-s0001.pdf]
